# Laminin α2 Chain-Deficiency is Associated with microRNA Deregulation in Skeletal Muscle and Plasma

**DOI:** 10.3389/fnagi.2014.00155

**Published:** 2014-07-03

**Authors:** Johan Holmberg, Azra Alajbegovic, Kinga Izabela Gawlik, Linda Elowsson, Madeleine Durbeej

**Affiliations:** ^1^Muscle Biology Unit, Department of Experimental Medical Science, University of Lund, Lund, Sweden

**Keywords:** fibrosis, inflammation, laminin, MDC1A, microRNA, muscular dystrophy

## Abstract

microRNAs (miRNAs) are widespread regulators of gene expression, but little is known of their potential roles in congenital muscular dystrophy type 1A (MDC1A). MDC1A is a severe form of muscular dystrophy caused by mutations in the gene encoding laminin α2 chain. To gain insight into the pathophysiological roles of miRNAs associated with MDC1A pathology, laminin α2 chain-deficient mice were evaluated by quantitative PCR. We demonstrate that expression of muscle-specific miR-1, miR-133a, and miR-206 is deregulated in laminin α2 chain-deficient muscle. Furthermore, expression of miR-223 and miR-21, associated with immune cell infiltration and fibrosis, respectively, is altered. Finally, we show that plasma levels of muscle-specific miRNAs are markedly elevated in laminin α2 chain-deficient mice and partially normalized in response to proteasome inhibition therapy. Altogether, our data suggest important roles for miRNAs in MDC1A pathology and we propose plasma levels of muscle-specific miRNAs as promising biomarkers for the progression of MDC1A.

## Introduction

Muscular dystrophy encompasses a group of inherited disorders mainly affecting skeletal muscle. Different types of muscular dystrophy are genetically diverse but share common phenotypic features including progressive myofiber degeneration, muscle weakness, and declined muscle function (Cohn and Campbell, 2000). Mutations in the human *LAMA2* gene, encoding the laminin α2 chain, lead to congenital muscular dystrophy type 1A (MDC1A). MDC1A is a severe form of muscular dystrophy characterized by hypotonia at birth, muscle weakness, delayed motor development, and joint contractures (Tome et al., 1994). Laminins are cruciform or T-shaped heterotrimeric molecules composed of one α, one β, and one γ subunit. To date, at least 18 different laminin isoforms have been identified. However, the most abundant laminin isoform in the skeletal muscle basement membrane is laminin-211 (α2, β1, and γ1) (Ehrig et al., 1990). Together with the dystrophin–glycoprotein complex laminin-211 forms a link between the basement membrane and the intracellular cytoskeleton protecting the muscle fiber from contraction-induced damage (Ervasti and Campbell, 1993).

Despite considerable research efforts there is currently no cure for MDC1A. Hence, the discovery that microRNAs (miRNAs) are deregulated in muscle diseases makes them attractive therapeutic targets (Eisenberg et al., 2007). miRNAs are small non-protein-coding RNAs, which regulate gene expression at the post-transcriptional level (Chen and Rajewsky, 2007). Investigations of miRNA expression and function in dystrophic muscles have identified a large number of deregulated miRNAs. Among the best characterized are three muscle-specific miRNAs: miR-1, miR-133a, and miR-206 (Chen et al., 2006; Yuasa et al., 2008). Moreover, miRNAs associated with specific features of muscular dystrophy such as infiltration of inflammatory cells (miR-223) and fibrogenesis (miR-21 and miR-29), are also altered in dystrophic muscles (Eisenberg et al., 2007; Greco et al., 2009). In addition, miRNAs are released into the blood stream of muscular dystrophy patients and mouse models, indicating that they represent biomarkers for disease progression and experimental therapies (Cacchiarelli et al., 2011; Mizuno et al., 2011).

An extended analysis of miRNA expression in MDC1A patients or mouse models has never been performed. Hence, in this study, we have analyzed expression of six miRNAs (miR-1, miR-133a, miR-206, miR-21, miR-29c, and miR-223) in muscle and plasma from two different MDC1A mouse models (*dy^3K^/dy^3K^* and *dy^2J^/dy^2J^*). The *dy^3K^/dy^3K^* mouse is completely deficient for laminin α2 and displays very severe muscle pathology whereas the *dy^2J^/dy^2J^* mouse, which expresses reduced levels of a truncated laminin α2, displays a milder muscular phenotype. Together, these mouse models are representative of the complexity of the MDC1A pathology.

Finally, our research group recently observed increased proteasomal activity in skeletal muscle from *dy^3K^/dy^3K^* mice, an observation that seems to be specific for muscular dystrophy caused by loss of laminin-211. Here, we have taken advantage of a proteasome inhibitor that reduces the *dy^3K^/dy^3K^* pathology (Körner et al., 2014), to investigate whether reduction in muscle pathology correlates with levels of muscle-specific miRNAs in plasma.

## Materials and Methods

### Animal models

Laminin α2 chain-deficient *dy^3K^/dy^3K^* mice have been described (Miyagoe et al., 1997). Wild-type and *dy^2J^/dy^2J^* (B6.WK-*Lama^2dy-2J^*/J) (Xu et al., 1994; Sunada et al., 1995) were purchased from Jackson laboratory and bred in the Biomedical Center vivarium in accordance with the animal care guidelines set by the Malmö/Lund (Sweden) ethical committee for animal research. Mice were analyzed at 6 weeks of age (*dy^2J^/dy^2J^*), 3 weeks of age (*dy^3K^/dy^3K^*), or at 9 days of age (young *dy^3K^/dy^3K^*). All comparisons were made against age-matched wild-type mice.

### RNA isolation

Skeletal muscle total RNA was extracted from quadriceps muscles snap-frozen in liquid nitrogen using the miRCURY RNA Isolation Kit following the manufacturer’s instructions (Exiqon). Blood was collected from heart puncture and transferred to anticoagulant tubes (EDTA) and centrifuged at 1100 × *g* for 10 min. Total RNA from blood plasma was extracted following the manufacturer’s instructions (Qiagen miRNeasy^®^ Mini Kit). Briefly, plasma was thawed on ice and centrifuged at 3000 × *g* for 5 min in a 4°C centrifuge. Fifty microliters of plasma per sample was transferred to a new microcentrifuge tube and 190 μl of QIAzol mixture containing 0.8 μg/μl MS2 bacteriophage RNA (Roche Applied Science) was added to each tube. Fifty microliters of chloroform was added to each tube followed by centrifugation at 12000 × *g* for 15 min in a 4°C microcentrifuge. The supernatant was transferred to a new microcentrifuge tube and 435 μl ethanol was added to each sample. A rinse step was performed with 1 × 500 μl RWT buffer and 3 × 500 μl RPE buffer. Total RNA was eluted by adding 50 μl RNase-free water to the membrane of the Qiagen RNeasy Mini spin column followed by centrifugation at 15000 × *g* for 1 min. The RNA was stored at −80°C.

### Quantitative RT-PCR

Twenty nanograms of muscle RNA was reverse transcribed using the miRCURY LNA Universal RT cDNA Synthesis Kit (Exiqon). The cDNA was diluted 80× and assayed in 10 μl PCR reactions according to the protocol for the miRCURY LNA Universal RT miRNA PCR system. The amplification was performed in a LightCycler 480 Real-Time PCR System (Roche) in 96-well plates. Primers were designed by Exiqon. Delta–delta Ct values were calculated relative to let-7a and miR-16 (Roberts et al., 2012).

One microliter of RNA blood plasma eluate was reverse transcribed in a 10 μl reaction using the miRCURY LNA Universal RT cDNA Synthesis Kit (Exiqon). The cDNA was diluted 40× and assayed in a 10 μl reaction according to the protocol for the miRCURY LNA™ Universal RT miRNA PCR system (Exiqon). miRNA plasma levels were calculated relative to miR-21a and miR-223 (Roberts et al., 2012).

No-RT control reactions were performed to ensure no DNA carryover. All amplifications were performed in triplicate on a LightCycler 480 Real-Time PCR System (Roche) in 96-well plates. The amplification curves were analyzed using the Roche LC software for determination of Cp (by second derivate method) and for melting curve analysis. The miRCURY RNA spike-in kit (synthetic control template) was used to control for the quality of the cDNA synthesis reaction. All oligonucleotide sequences were designed by and ordered from Exiqon with the following product numbers: hsa-miR-1, 204344; hsa-let-7a-5p, 204775; hsa-miR-16-5p, 204409; hsa-miR-21-5p, 204230; hsa-miR-29c-3p, 204729; hsa-miR-133a, 204788; hsa-miR-206, 204616; and hsa-miR-223-3p, 204256.

### Histology

Quadriceps muscles were isolated and frozen in OCT (Tissue Tek) in liquid nitrogen. Transverse cryosections of 8 μm were transferred to positively charged glass slides and stored in −80°C. Sections were stained with hematoxylin and eosin (H&E) for quantification of centrally nucleated fibers or with Sirius red and Fast green (Sigma-Aldrich) for visualization of collagenous and non-collagenous tissue, respectively. Central nucleation is represented as a percentage of the total number of fibers counted in entire transverse quadriceps sections. For Sirius red and Fast green staining, sections were acclimated to RT for 15 min and fixated in Bouin’s solution at 55°C for 1 h, incubated in 0.1% Fast green for 10 min followed by incubation in 0.1% Picro Sirius red for 30 min. Sections were then dehydrated in ethanol and cleared in xylene.

### Immunofluorescence assays

Transverse sections of 8 μm were fixed in ice-cold acetone for 8 min. For CD11b (rat monoclonal M1/70, 1:300, BD Pharmingen), CD68 (rat monoclonal FA-11, 1:100, AbD Serotec), and laminin γ1 chain (rabbit polyclonal 1083 + E1, 1:100, kindly provided by Dr. T. Sasaki), sections were blocked in 3% BSA in PBS at RT for 30 min followed by incubation in primary antibody at RT for 1 h. For embryonic myosin heavy chain (mouse monoclonal F1.652, 1:10, Developmental Studies Hybridoma Bank), sections were blocked in 4% goat serum and 0.05% Triton-X in PBS at RT for 40 min followed by incubation in primary antibody at RT for 90 min. Primary antibodies were incubated with appropriate secondary antibodies for 60 or 45 min (embryonic myosin heavy chain). Antibody stained sections were analyzed using a Zeiss Axioplan fluorescent microscope using Openlab 3 and an ORCA 1394 ER digital camera. The percentage of embryonic myosin heavy chain–positive fibers was obtained by counting the number of fibers positive for embryonic myosin heavy chain in a whole quadriceps section and dividing by the total number of myofibers.

### Hydroxyproline assay (collagen content in muscle)

Quadriceps muscles were isolated and frozen in liquid nitrogen. Samples were weighed and incubated overnight in 200 μl concentrated HCl (12 M) at 95°C. Twenty five microliters of hydrolyzate was neutralized with 25 μl NaOH (0.6 M) and incubated with 450 μl Chloramine-T reagent (0.056 M) at RT for 25 min. A volume of 500 μl freshly prepared Ehrlich’s reagent [1 M 4-(dimethylamino)benzaldehyde] was added to each sample and incubated at 65°C for 1 h. After cooling on ice, 100 μl in duplicates was transferred to a 96-well plate and absorbance was read at 560 nm. Standards from 4-hydroxyproline at concentrations (microgram per milliliter); 0, 0.05, 0.1, 0.15, 0.2, 0.25, 0.4 were treated the same way as the samples. Absorbance (*A*_560_) of standards was plotted against amount of hydroxyproline (microgram) and a linear regression was performed to determine slope and intercept. All absorbance values were subtracted with blank (0 μg/ml hydroxyproline). Content of hydroxyproline in samples was calculated by equation:

$$x\;\left(\mu \text{g}\right)=\left(A_{560}-Y_{\text{axis}\;\text{intercept}}\right)\;∕\text{slope}$$

Collagen conversion factor = 13.5 (Neuman and Logan, 1950). Values are presented as relative amount of collagen.

### Creatine kinase assay

Blood was collected from heart puncture and transferred to anticoagulant tubes (EDTA) and centrifuged at 1100 × *g* for 10 min at 4°C. Plasma was analyzed at Clinical Chemistry Laboratory at Skåne University Hospital. The CK_P_S Cobas method was used to quantify enzyme activity.

### Bortezomib treatment

Briefly, *dy^3K^/dy^3K^* mice were administered 0.4 mg/kg bortezomib (LC Laboratories) via tail vein injection at 2.5 and 3.5 weeks of age. Mice were analyzed 14 days after injection (Körner et al., 2014).

### Statistical analyses

Data shown in qRT-PCR analyses are the result of at least three independent experiments. Statistical significance of differences between means was assessed by one-way analysis of variance. Multiple comparisons were performed using the Holm–Sidak method. Unpaired *t*-test was used when two groups were compared. *P* < 0.05 was considered significant. All statistical analysis was performed using PRISM 6.0b software (GraphPad).

## Results

### Altered expression of muscle-specific miRNAs in *dy*^*3K*^/*dy*^*3K*^ and *dy*^*2J*^/*dy*^*2J*^ quadriceps muscle

To determine whether miRNAs previously shown to be differentially expressed in dystrophic muscle (Eisenberg et al., 2007; Greco et al., 2009) are deregulated in MDC1A, we investigated miRNA expression in two different mouse models: *dy^3K^/dy^3K^* (completely devoid of laminin α2 chain) and *dy^2J^/dy^2J^* mice (expressing slightly reduced levels of a truncated laminin α2 chain) (Xu et al., 1994; Miyagoe et al., 1997). The *dy^3K^/dy^3K^* mice were analyzed at 3 weeks of age due to lethality between 4 and 5 weeks of age. The *dy^2J^/dy^2J^* mice however do not display any obvious signs of muscle pathology at that time point. Hence, these mice were instead analyzed at 6 weeks of age correlating with clear signs of muscle pathology.

**Figure 1:**
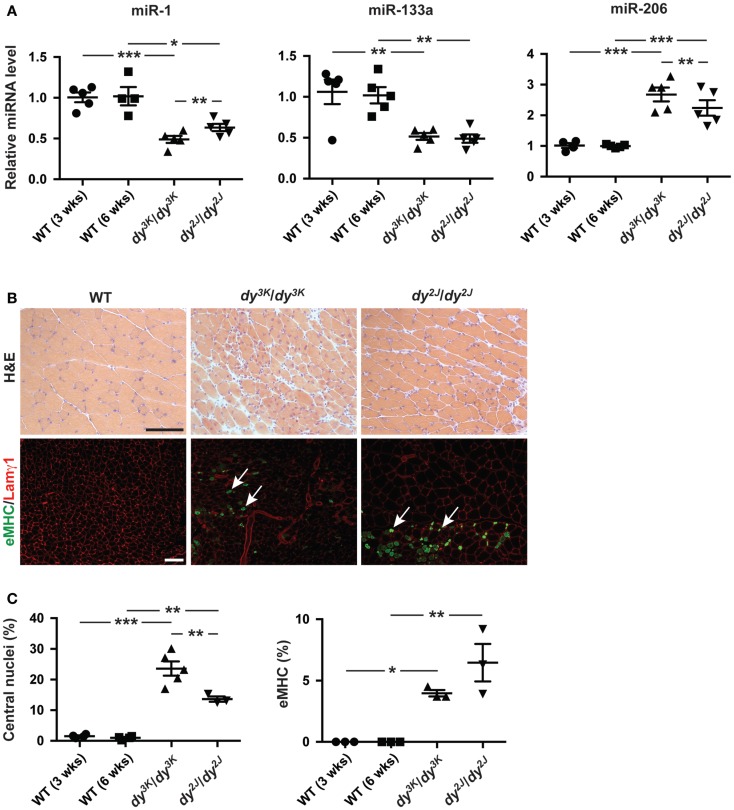
**Laminin α2 chain-deficiency results in altered expression of muscle-specific miRNAs**. **(A)** RT-qPCR analysis of indicated miRNAs in muscles from 3-week-old *dy^3K^/dy^3K^* and 6-week-old *dy^2J^/dy^2J^* mice (*n* ≥ 4). **(B)** Transverse sections of muscles from WT, *dy^3K^/dy^3K^*, and *dy^2J^/dy^2J^* mice stained for H&E to visualize histopathology and eMHC/Lamγ1 to identify early regenerating fibers. Arrows indicate eMHC positive cells (green). Bar, 100 μm. **(C)** Percentage of fibers with centralized nuclei or positive for eMHC in muscles from WT, *dy^3K^/dy^3K^*, and *dy^2J^/dy^2J^* mice (*n* ≥ 3). WT, wild-type; H&E, hematoxylin/eosin; eMHC, embryonic myosin heavy chain; Lamγ1, laminin γ1 chain. Error bars represent SEM, *P < 0.05, **P < 0.01, ***P < 0.001.

We observed a decreased expression of miR-1 and miR-133a and an increase in miR-206 expression in *dy^3K^/dy^3K^* and *dy^2J^/dy^2J^* quadriceps muscle compared with wild-type controls (Figure 1A). Moreover, both miR-1 and miR-206 were differentially expressed in *dy^3K^/dy^3K^* and *dy^2J^/dy^2J^* mice, which may reflect differences in disease development. The observation that miR-206 expression was significantly higher in muscle from *dy^3K^/dy^3K^* mice compared with *dy^2J^/dy^2J^*, together with the established role of miR-206 in muscle regeneration prompted us to investigate the number of myofibers with central nuclei (reflecting overall muscle regeneration) and the number of fibers expressing embryonic myosin heavy chain (eMHC, reflecting the initial phase of myofiber regeneration) in *dy^3K^/dy^3K^* and *dy^2J^/dy^2J^* mice (Yuasa et al., 2008; Liu et al., 2012). We observed a significant increase in overall myofiber regeneration in *dy^3K^/dy^3K^* compared with *dy^2J^/dy^2J^* mice, closely resembling miR-206 expression (Figures 1A–C). However, staining against eMHC, which is transiently expressed in nascent myofibers revealed a low number of positive fibers, both in *dy^3K^/dy^3K^* and *dy^2J^/dy^2J^* mice, indicating that miR-206 expression reflects the overall regenerative status of the muscle rather than the initial stages of regeneration (Figures 1B,C). These findings are consistent with the effect of induced muscle damage on miR-206 expression, which increased markedly on day 5 post-injury (Yuasa et al., 2008).

Taken together, miR-1 and miR-133a are significantly downregulated in *dy^3K^/dy^3K^* and *dy^2J^/dy^2J^* muscle while miR-206 expression is upregulated, reflecting the overall regenerative status of the dystrophic muscle.

### Expression of miR-21 is deregulated in *dy*^*3K*^/*dy*^*3K*^ and *dy*^*2J*^/*dy*^*2J*^ quadriceps muscle

**Figure 2:**
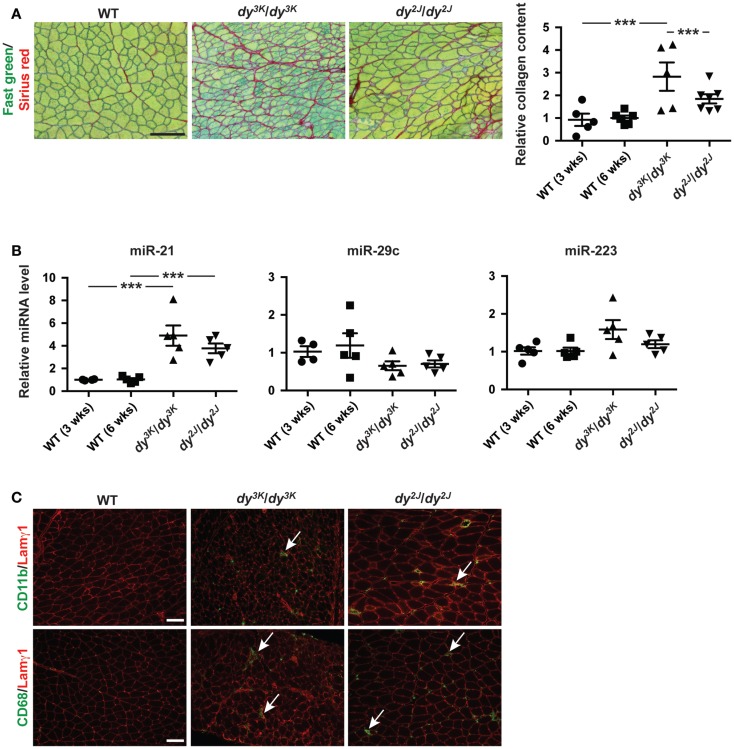
**Fibrosis in laminin α2 chain-deficient muscle is associated with increased expression of miR-21**. **(A)** Left: transverse sections of muscles from WT, *dy^3K^/dy^3K^*, and *dy^2J^/dy^2J^* mice stained for Fast green/Sirius red to visualize non-collagenous and collagenous tissue, respectively. Bar, 100 μm. Right: quantification of relative collagen content (fibrosis) in *dy^3K^/dy^3K^* and *dy^2J^/dy^2J^* mice (*n* ≥ 5). **(B)** RT-qPCR analysis of indicated miRNAs in muscles from *dy^3K^/dy^3K^* and *dy^2J^/dy^2J^* mice (*n* ≥ 4). **(C)** CD11b/CD68/Lamγ1 stained sections of muscles from WT, *dy^3K^/dy^3K^*, and *dy^2J^/dy^2J^* mice. Arrows indicate CD11b and CD68-positive cells (green). Bar, 100 μm. WT, wild-type; Lamγ1, laminin γ1 chain. Error bars represent SEM, ***P < 0.001.

Dystrophic myofibers are progressively replaced by adipose and fibrotic tissue leading to irreversible loss of muscle (Mann et al., 2011). Laminin α2 chain-deficient muscles display extensive fibrosis, both in MDC1A patients and in *dy^3K^/dy^3K^* and *dy^2J^/dy^2J^* mice as shown by Fast green and Sirius red staining (visualizing non-collagenous and collagenous tissue, respectively) and biochemical collagen quantification (Figure 2A). Newly published data demonstrate a role of miR-21 and miR-29 as regulators of fibrogenesis (Ardite et al., 2012; Wang et al., 2012).

Based on these observations we analyzed the expression of miR-21 and miR-29c in muscle from *dy^3K^/dy^3K^* and *dy^2J^/dy^2J^* mice. We noticed a significant upregulation of miR-21 in both *dy^3K^/dy^3K^* and *dy^2J^/dy^2J^* mice compared with wild-type controls (Figure 2B). However, we did not observe any significant difference in miR-29c expression. Previous data suggest that miR-29c expression in dystrophic muscle depends on muscle group and age (Greco et al., 2009; Roberts et al., 2012). It may be that the effect of laminin α2 chain-deficiency on miR-29c expression is influenced by similar factors.

Dystrophic muscles are characterized by infiltration of inflammatory cells (Mann et al., 2011). This is also true for laminin α2 chain-deficient muscle (Pegoraro et al., 1996). miR-223 has been shown to be involved in granulocyte production and several studies report increased expression of miR-223 in dystrophic muscle (Eisenberg et al., 2007; Johnnidis et al., 2008). We did not observe any significant upregulation of miR-223 in quadriceps muscles from *dy^3K^/dy^3K^* or *dy^2J^/dy^2J^* mice at 3 and 6 weeks of age, respectively (Figure 2B). This is consistent with the relatively low number of CD11b- and CD68-positive immune cells (monocytes/macrophages) at the indicated time points (Figure 2C).

### Circulating muscle-specific miRNAs are enriched in *dy*^*3K*^/*dy*^*3K*^ and *dy*^*2J*^/*dy*^*2J*^ mice

**Figure 3:**
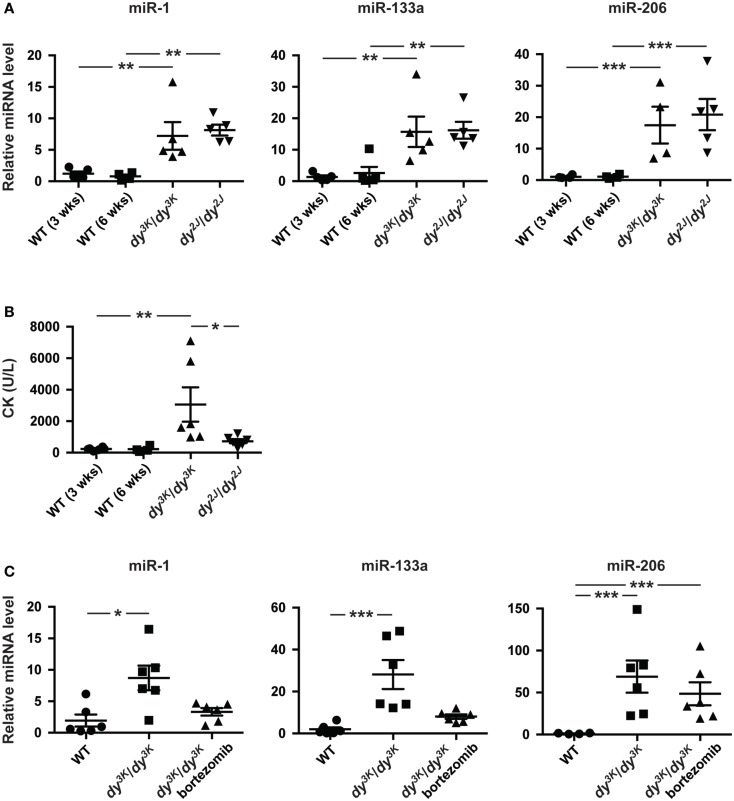
**Enrichment of muscle-specific miRNAs in plasma upon laminin α2-deficiency**. **(A)** RT-qPCR analysis of plasma levels of indicated miRNAs in 3-week-old *dy^3K^/dy^3K^* and 6-week-old *dy^2J^/dy^2J^* mice (*n* ≥ 4). **(B)** Analysis of CK levels in plasma from WT, *dy^3K^/dy^3K^*, and *dy^2J^/dy^2J^* mice (*n* ≥ 4). **(C)** RT-qPCR analysis of plasma levels of indicated miRNAs in WT, *dy^3K^/dy^3K^*, and bortezomib injected *dy^3K^/dy^3K^* mice (*n* ≥ 4). WT, wild-type; CK, creatine kinase. Error bars represent SEM, *P < 0.05, **P < 0.01, ***P < 0.001.

In addition to aberrant miRNA expression in skeletal muscle, muscular dystrophy patients and mice display altered levels of miRNAs in the blood (Cacchiarelli et al., 2011; Mizuno et al., 2011; Vignier et al., 2013). Hence, we investigated levels of muscle-specific miR-1, miR-133a, and miR-206 in plasma from 3-week-old *dy^3K^/dy^3K^* and 6-week-old *dy^2J^/dy^2J^* mice. We observed a significant increase in miR-1 (~7-fold), miR-133a (~15-fold), and miR-206 (~15-fold) compared with wild-type controls, however no significant differences were observed between *dy^3K^/dy^3K^* and *dy^2J^/dy^2J^* mice (Figure 3A).

Recent data suggest that extra-cellular miRNAs is the result of selective rather than uncontrolled release from damaged myofibers (Roberts et al., 2013). To investigate this further we analyzed plasma creatine kinase (CK) levels, a classical index of sarcolemmal integrity. Significant increase in CK levels was observed only in *dy^3K^/dy^3K^* mice (Figure 3B). Notably, the degree of sarcolemmal damage between *dy^3K^/dy^3K^* and *dy^2J^/dy^2J^* mice is in sharp contrast to levels of muscle-specific miRNAs in circulation, supporting the observation that release of miRNAs into circulation is a regulated process rather than simple leakage from damaged fibers. Taken together, the abundance of circulating muscle-specific miRNAs is significantly increased upon laminin α2 chain-deficiency.

Increased proteasomal activity is a feature of MDC1A and recent studies demonstrated that proteasome inhibition partially improves muscle integrity in *dy^3K^/dy^3K^* mice accompanied by increased expression of miR-1 and miR-133a (Carmignac et al., 2011; Körner et al., 2014). To determine if reduced muscle pathology had an impact on plasma levels of dysregulated miRNAs, *dy^3K^/dy^3K^* mice were given bortezomib (a proteasome inhibitor). Notably, administration of bortezomib resulted in a partial normalization of plasma levels of miR-1 and miR-133a in *dy^3K^/dy^3K^* mice (Figure 3C). However, bortezomib did not affect miR-206 plasma levels. This is consistent with observations that bortezomib does not significantly reduce myofiber regeneration in *dy^3K^/dy^3K^* mice (Körner et al., 2014). In summary, the partial normalization of miR-1 and miR-133a in response to bortezomib administration indicates that these miRNAs are promising disease biomarkers for MDC1A.

### miRNA expression in *dy*^*3K*^/*dy*^*3K*^ mice changes dynamically over time

**Figure 4:**
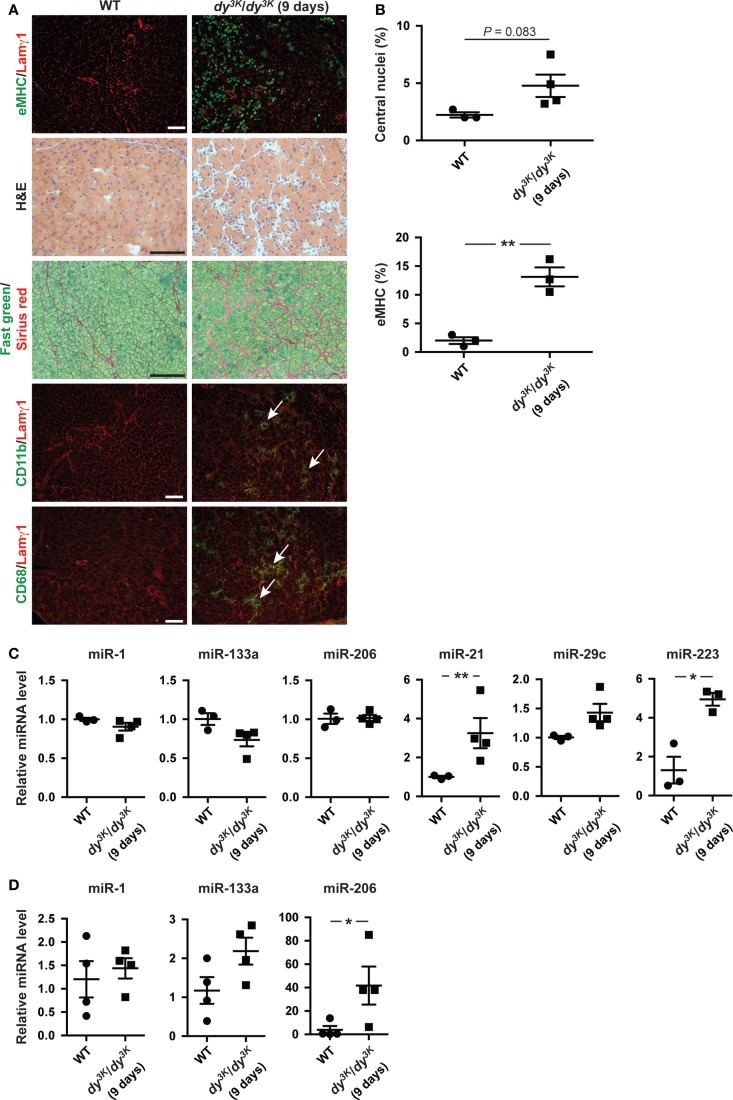
**Dynamic miRNA expression in laminin α2-deficient mice over time**. **(A)** Transverse sections of muscles from 9-day-old WT and *dy^3K^/dy^3K^* mice stained for eMHC/Lamγ1, H&E, Fast green/Sirius red, and CD11b/CD68/Lamγ1. Arrows indicate CD11b and CD68-positive cells (green). Bar, 100 μm. **(B)** Percentage of fibers with centralized nuclei or positive for eMHC in muscles from WT and *dy^3K^/dy^3K^* mice (*n* ≥ 3). **(C)** RT-qPCR analysis of indicated miRNAs in muscles from 9-day-old WT and *dy^3K^/dy^3K^* mice (*n* ≥ 3). **(D)** RT-qPCR analysis of plasma levels of indicated miRNAs in 9-day-old WT and *dy^3K^/dy^3K^* mice (*n* = 4). WT, wild-type; H&E, hematoxylin/eosin; eMHC, embryonic myosin heavy chain; Lamγ1, laminin γ1 chain. Error bars represent SEM, *P < 0.05, **P < 0.01.

microRNA expression is a dynamic process possibly reflecting the development of the underlying dystrophic pathology (Roberts et al., 2013). Hence, we analyzed the expression of miRNAs in *dy^3K^/dy^3K^* muscles and plasma at an early age (9 days of age). At this time point, the number of regenerating fibers positive for eMHC is high while the level of overall myofiber regeneration is low, reflected in unaltered expression of miR-206 in muscle (Figures 4A–C). In addition, at 9 days of age *dy^3K^/dy^3K^* mice display extensive infiltration of inflammatory cells and accumulation of extra-cellular matrix components (Figure 4A). Accordingly, we noticed a significant increase in miR-223 (immune cells) and miR-21 expression (fibrosis) in muscle from *dy^3K^/dy^3K^* mice compared with wild-type controls (Figure 4C). Notably, expression of muscle-specific miR-1 and miR-133a were unaffected at young ages (Figure 4C).

We also investigated levels of circulating muscle-specific miRNAs in young *dy^3K^/dy^3K^* mice. Extra-cellular levels of miR-1 and miR-133a were unaltered whereas levels of miR-206 were significantly increased compared with wild-type controls (in contrast to muscle) (Figures 4C,D). The discrepancy between muscle and plasma levels of miR-206 could be due to plasma levels preceding the increase in miR-206 expression in muscle. It is also possible that additional muscle groups contribute to the increase in miR-206 plasma levels (Roberts et al., 2013). Taken together, abundance of miRNAs in *dy^3K^/dy^3K^* mice is dynamic and changes as the muscular dystrophy develops.

## Discussion

In the current study, we present evidences that MDC1A physiopathology is associated with altered expression of miRNAs, both in muscle and plasma. Specifically, we demonstrate that loss of laminin α2 chain leads to downregulation of muscle-specific miR-1 and miR-133a together with increased expression of miR-206 in muscle, consistent with data on other types of muscular dystrophy. The role of miR-206 in myofiber regeneration is well characterized and loss of miR-206 leads to delayed regeneration upon induced muscle damage (Liu et al., 2012). In contrast, the precise function of miR-1 and miR-133a in skeletal muscle is less clear. Studies on C2C12 myoblasts suggest that miR-133a and miR-1 promote proliferation and differentiation, respectively (Chen et al., 2006). However, mice deficient for miR-133a do not display any skeletal muscle anomalies until they are adult and skeletal muscle from miR-1-deficient mice is grossly normal (Zhao et al., 2007; Liu et al., 2011). These observations make it difficult to draw any firm conclusions regarding the impact of miR-133a and miR-1 dysregulation on the MDC1A pathology.

In this report, we also describe that miR-223 and miR-21 expression is positively associated with inflammation and fibrosis, respectively, in *dy^3K^/dy^3K^* and *dy^2J^/dy^2J^* muscle. Inflammation in MDC1A muscles ultimately results in progressive and irreversible replacement of muscle by adipose and fibrotic tissue. Severe inflammation in muscle from *dy^3K^/dy^3K^* mice is evident already at 7 days of age (Gawlik et al., 2014). However, inflammation appears arrested in older muscles (Figures 2C and 4A) (Gawlik et al., 2014). Importantly, we show that miR-223 expression reflects the degree of immune cell infiltration in muscle from *dy^3K^/dy^3K^* mice and could therefore be involved in modulating the inflammatory response in MDC1A. The importance of immune cells in chronic myopathic conditions is emphasized by experiments on dystrophin-deficient *mdx* mice lacking functional B and/or T lymphocytes. These mice displayed reduced levels of the profibrotic cytokine transforming growth factor (TGF)-β and less diaphragm fibrosis (Morrison et al., 2000; Farini et al., 2007).

Furthermore, muscle fibrosis is associated with disease progression in MDC1A. Despite efforts to combat fibrogenesis in laminin α2-deficient mice, clinical applications for MDC1A remain years away (Elbaz et al., 2012; Meinen et al., 2012). Hence, the recent discovery that miR-21 and miR-29c can function as antifibrotic molecules in dystrophic muscles suggests miRNAs as attractive therapeutic candidates (Cacchiarelli et al., 2010; Ardite et al., 2012; Wang et al., 2012). In contrast to miR-21, we did not observe altered expression of miR-29c in *dy^3K^/dy^3K^* and *dy^2J^/dy^2J^* muscle. Expression of miR-29c is downregulated by TGF-β signaling, which is increased in dystrophic skeletal muscle (Bernasconi et al., 1995, 1999). However, TGF-β signaling seems to be less pronounced in MDC1A muscle, which may explain the unaltered expression of miR-29c in *dy^3K^/dy^3K^* and *dy^2J^/dy^2J^* muscle (Bernasconi et al., 1999). Collectively, these data suggest that fibrosis in MDC1A may be driven by other molecules than TGF-β.

It should be noted that Roberts and colleagues demonstrated important differences in miRNA expression between individual muscle groups in the *mdx* mouse (Roberts et al., 2012). In this report, miRNA expression analysis was limited to quadriceps muscles. Future studies on miRNA expression in laminin α2-deficient mice should include additional skeletal muscle groups.

Finally, we demonstrate that MDC1A is accompanied by increased levels of circulating muscle-specific miRNAs, which are partially normalized upon reduction of the dystrophic pathology. The precise biological function for extra-cellular miRNA remains largely unknown. However, we and others have demonstrated that expression of muscle-specific miRNAs in muscle does not reflect miRNA abundance in plasma or serum, indicating that miRNAs could enter circulation by exocytosis or be released in vesicles rather than by uncontrolled leakage. Moreover, the enrichment of circulating muscle-specific miRNAs in laminin α2-deficient mice is similar to observations in the *mdx* mouse, despite significantly more sarcolemmal damage in the latter (Straub et al., 1997; Roberts et al., 2012).

We also demonstrate that levels of plasma miRNAs in *dy^3K^/dy^3K^* change over time. These observations are consistent with data from other muscle disorders and suggest that levels of circulating miRNAs could serve as biomarkers for monitoring treatment strategies and diagnosis of MDC1A. Current methods are largely based on muscle biopsies and CK assays. In contrast to CK, extra-cellular miRNAs are resistant to stress and more accurately reflects disease severity (Cacchiarelli et al., 2011; Mizuno et al., 2011). In addition, miRNAs are present in numerous biological fluids easily accessible for analysis, including saliva and urine (De Guire et al., 2013). However, we observed a significant variability in levels of muscle-specific miRNAs in plasma from *dy^3K^/dy^3K^* and *dy^2J^/dy^2J^* mice, similar to studies on *mdx* mice (Cacchiarelli et al., 2011; Roberts et al., 2012).

A possible reason is that dystrophic muscles typically enter cycles of myofiber degeneration/regeneration, which lead to irreversible muscle wasting over time. Variation in onset of these regeneration cycles between mice could at least explain the variability in miR-206 levels in circulation. To establish miRNAs as biomarkers in MDC1A pathology, additional studies identifying and coupling miRNA levels in biofluids to pathology markers need to be performed. Moreover, global analysis of extra-cellular miRNA levels in laminin α2-deficient animals should facilitate the identification of miRNA profiles that correlate well with degree of muscle pathology.

Taken together, the discovery that miRNA expression is altered in MDC1A mouse models opens new strategies to combat this devastating disorder. Despite several challenges, recent refinements in delivery carriers, miRNA mimic molecules, and anti-miRs have improved delivery, specificity, and stability of miRNA therapeutics. Currently, one miRNA drug (miravirsen) has reached clinical trials and additional miRNA drugs are likely to enter clinical trials soon (Gebert et al., 2014).

## Author Contributions

Johan Holmberg and Madeleine Durbeej designed the experiments. Johan Holmberg and Azra Alajbegovic performed most of the experiments. Kinga Izabela Gawlik designed and analyzed immunofluorescence assays. Linda Elowsson performed and analyzed hydroxyproline assays. Johan Holmberg wrote the paper and other authors commented on the manuscript.

## Conflict of Interest Statement

The authors declare that the research was conducted in the absence of any commercial or financial relationships that could be construed as a potential conflict of interest.

## References

[B1] Ardite E. Perdiguero E. Vidal B. Gutarra S. Serrano A. L. Munoz-Canoves P. (2012). PAI-1-regulated miR-21 defines a novel age-associated fibrogenic pathway in muscular dystrophy. J. Cell Biol. 196, 163–175 10.1083/jcb.201105013 22213800 PMC3255978

[B2] Bernasconi P. Di Blasi C. Mora M. Morandi L. Galbiati S. Confalonieri P. (1999). Transforming growth factor-beta1 and fibrosis in congenital muscular dystrophies. Neuromuscul. Disord. 9, 28–33 10.1016/S0960-8966(98)00093-5 10063832

[B3] Bernasconi P. Torchiana E. Confalonieri P. Brugnoni R. Barresi R. Mora M. (1995). Expression of transforming growth factor-beta 1 in dystrophic patient muscles correlates with fibrosis. Pathogenetic role of a fibrogenic cytokine. J. Clin. Invest. 96, 1137–1144 10.1172/JCI118101 7635950 PMC185304

[B4] Cacchiarelli D. Legnini I. Martone J. Cazzella V. D’Amico A. Bertini E. (2011). miRNAs as serum biomarkers for Duchenne muscular dystrophy. EMBO Mol. Med. 3, 258–265 10.1002/emmm.201100133 21425469 PMC3112257

[B5] Cacchiarelli D. Martone J. Girardi E. Cesana M. Incitti T. Morlando M. (2010). microRNAs involved in molecular circuitries relevant for the Duchenne muscular dystrophy pathogenesis are controlled by the dystrophin/nNOS pathway. Cell Metab. 12, 341–351 10.1016/j.cmet.2010.07.008 20727829

[B6] Carmignac V. Quere R. Durbeej M. (2011). Proteasome inhibition improves the muscle of laminin alpha2 chain-deficient mice. Hum. Mol. Genet. 20, 541–552 10.1093/hmg/ddq499 21084425

[B7] Chen J. F. Mandel E. M. Thomson J. M. Wu Q. Callis T. E. Hammond S. M. (2006). The role of microRNA-1 and microRNA-133 in skeletal muscle proliferation and differentiation. Nat. Genet. 38, 228–233 10.1038/ng1725 16380711 PMC2538576

[B8] Chen K. Rajewsky N. (2007). The evolution of gene regulation by transcription factors and microRNAs. Nat. Rev. Genet. 8, 93–103 10.1038/nrg1990 17230196

[B9] Cohn R. D. Campbell K. P. (2000). Molecular basis of muscular dystrophies. Muscle Nerve 23, 1456–1471 10.1002/1097-4598(200010)23:10<1456::AID-MUS2>3.0.CO;2-T 11003781

[B10] De Guire V. Robitaille R. Tetreault N. Guerin R. Menard C. Bambace N. (2013). Circulating miRNAs as sensitive and specific biomarkers for the diagnosis and monitoring of human diseases: promises and challenges. Clin. Biochem. 46, 846–860 10.1016/j.clinbiochem.2013.03.015 23562576

[B11] Ehrig K. Leivo I. Argraves W. S. Ruoslahti E. Engvall E. (1990). Merosin, a tissue-specific basement membrane protein, is a laminin-like protein. Proc. Natl. Acad. Sci. U.S.A. 87, 3264–3268 10.1073/pnas.87.9.3264 2185464 PMC53880

[B12] Eisenberg I. Eran A. Nishino I. Moggio M. Lamperti C. Amato A. A. (2007). Distinctive patterns of microRNA expression in primary muscular disorders. Proc. Natl. Acad. Sci. U.S.A. 104, 17016–17021 10.1073/pnas.0708115104 17942673 PMC2040449

[B13] Elbaz M. Yanay N. Aga-Mizrachi S. Brunschwig Z. Kassis I. Ettinger K. (2012). Losartan, a therapeutic candidate in congenital muscular dystrophy: studies in the dy(2J)/dy(2J) mouse. Ann. Neurol. 71, 699–708 10.1002/ana.22694 22522482

[B14] Ervasti J. M. Campbell K. P. (1993). A role for the dystrophin-glycoprotein complex as a transmembrane linker between laminin and actin. J. Cell Biol. 122, 809–823 10.1083/jcb.122.4.809 8349731 PMC2119587

[B15] Farini A. Meregalli M. Belicchi M. Battistelli M. Parolini D. D’Antona G. (2007). T and B lymphocyte depletion has a marked effect on the fibrosis of dystrophic skeletal muscles in the scid/mdx mouse. J. Pathol. 213, 229–238 10.1002/path.2213 17668421

[B16] Gawlik K. I. Holmberg J. Durbeej M. (2014). Loss of dystrophin and beta-sarcoglycan, respectively, significantly exacerbates the phenotype of laminin alpha2 chain-deficient animals. Am. J. Pathol. 184, 740–752 10.1016/j.ajpath.2013.11.017 24393714

[B17] Gebert L. F. Rebhan M. A. Crivelli S. E. Denzler R. Stoffel M. Hall J. (2014). Miravirsen (SPC3649) can inhibit the biogenesis of miR-122. Nucleic Acids Res. 42, 609–621 10.1093/nar/gkt852 24068553 PMC3874169

[B18] Greco S. De Simone M. Colussi C. Zaccagnini G. Fasanaro P. Pescatori M. (2009). Common micro-RNA signature in skeletal muscle damage and regeneration induced by Duchenne muscular dystrophy and acute ischemia. FASEB J. 23, 3335–3346 10.1096/fj.08-128579 19528256

[B19] Johnnidis J. B. Harris M. H. Wheeler R. T. Stehling-Sun S. Lam M. H. Kirak O. (2008). Regulation of progenitor cell proliferation and granulocyte function by microRNA-223. Nature 451, 1125–1129 10.1038/nature06607 18278031

[B20] Körner Z. Holmberg J. Fontes-Oliveira C. C. Carmignac V. Durbeej M. (2014). Bortezomib partially improves laminin α2 chain-deficient muscular dystrophy. Am. J. Pathol. 184, 1518–1528 10.1016/j.ajpath.2014.01.019 24631023

[B21] Liu N. Bezprozvannaya S. Shelton J. M. Frisard M. I. Hulver M. W. McMillan R. P. (2011). Mice lacking microRNA 133a develop dynamin 2-dependent centronuclear myopathy. J. Clin. Invest. 121, 3258–3268 10.1172/JCI46267 21737882 PMC3148737

[B22] Liu N. Williams A. H. Maxeiner J. M. Bezprozvannaya S. Shelton J. M. Richardson J. A. (2012). microRNA-206 promotes skeletal muscle regeneration and delays progression of Duchenne muscular dystrophy in mice. J. Clin. Invest. 122, 2054–2065 10.1172/JCI62656 22546853 PMC3366415

[B23] Mann C. J. Perdiguero E. Kharraz Y. Aguilar S. Pessina P. Serrano A. L. (2011). Aberrant repair and fibrosis development in skeletal muscle. Skelet. Muscle 1, 21 10.1186/2044-5040-1-21 21798099 PMC3156644

[B24] Meinen S. Lin S. Ruegg M. A. (2012). Angiotensin II type 1 receptor antagonists alleviate muscle pathology in the mouse model for laminin-alpha2-deficient congenital muscular dystrophy (MDC1A). Skelet. Muscle 2, 18 10.1186/2044-5040-2-18 22943509 PMC3598380

[B25] Miyagoe Y. Hanaoka K. Nonaka I. Hayasaka M. Nabeshima Y. Arahata K. (1997). Laminin alpha2 chain-null mutant mice by targeted disruption of the Lama2 gene: a new model of merosin (laminin 2)-deficient congenital muscular dystrophy. FEBS Lett. 415, 33–39 10.1016/S0014-5793(97)01007-7 9326364

[B26] Mizuno H. Nakamura A. Aoki Y. Ito N. Kishi S. Yamamoto K. (2011). Identification of muscle-specific microRNAs in serum of muscular dystrophy animal models: promising novel blood-based markers for muscular dystrophy. PLoS ONE 6:e18388 10.1371/journal.pone.0018388 21479190 PMC3068182

[B27] Morrison J. Lu Q. L. Pastoret C. Partridge T. Bou-Gharios G. (2000). T-cell-dependent fibrosis in the mdx dystrophic mouse. Lab. Invest. 80, 881–891 10.1038/labinvest.3780092 10879739

[B28] Neuman R. E. Logan M. A (1950). The determination of hydroxyproline. J. Biol. Chem. 184, 299–306 15421999

[B29] Pegoraro E. Mancias P. Swerdlow S. H. Raikow R. B. Garcia C. Marks H. (1996). Congenital muscular dystrophy with primary laminin alpha2 (merosin) deficiency presenting as inflammatory myopathy. Ann. Neurol. 40, 782–791 10.1002/ana.410400515 8957020

[B30] Roberts T. C. Blomberg K. E. McClorey G. Andaloussi S. E. Godfrey C. Betts C. (2012). Expression analysis in multiple muscle groups and serum reveals complexity in the microRNA transcriptome of the mdx mouse with implications for therapy. Mol. Ther. Nucleic Acids 1, e39 10.1038/mtna.2012.26 23344181 PMC3437806

[B31] Roberts T. C. Godfrey C. McClorey G. Vader P. Briggs D. Gardiner C. (2013). Extracellular microRNAs are dynamic non-vesicular biomarkers of muscle turnover. Nucleic Acids Res. 41, 9500–9513 10.1093/nar/gkt724 23945935 PMC3814379

[B32] Straub V. Rafael J. A. Chamberlain J. S. Campbell K. P. (1997). Animal models for muscular dystrophy show different patterns of sarcolemmal disruption. J. Cell Biol. 139, 375–385 10.1083/jcb.139.2.375 9334342 PMC2139791

[B33] Sunada Y. Bernier S. M. Utani A. Yamada Y. Campbell K. P. (1995). Identification of a novel mutant transcript of laminin alpha 2 chain gene responsible for muscular dystrophy and dysmyelination in dy2J mice. Hum. Mol. Genet. 4, 1055–1061 10.1093/hmg/4.6.1055 7655459

[B34] Tome F. M. Evangelista T. Leclerc A. Sunada Y. Manole E. Estournet B. (1994). Congenital muscular dystrophy with merosin deficiency. C. R. Acad. Sci. III, Sci. Vie 317, 351–357 8000914

[B35] Vignier N. Amor F. Fogel P. Duvallet A. Poupiot J. Charrier S. (2013). Distinctive serum miRNA profile in mouse models of striated muscular pathologies. PLoS ONE 8:e55281 10.1371/journal.pone.0055281 23418438 PMC3572119

[B36] Wang L. Zhou L. Jiang P. Lu L. Chen X. Lan H. (2012). Loss of miR-29 in myoblasts contributes to dystrophic muscle pathogenesis. Mol. Ther. 20, 1222–1233 10.1038/mt.2012.35 22434133 PMC3369280

[B37] Xu H. Wu X. R. Wewer U. M. Engvall E. (1994). Murine muscular dystrophy caused by a mutation in the laminin alpha 2 (Lama2) gene. Nat. Genet. 8, 297–302 10.1038/ng1194-297 7874173

[B38] Yuasa K. Hagiwara Y. Ando M. Nakamura A. Takeda S. Hijikata T. (2008). microRNA-206 is highly expressed in newly formed muscle fibers: implications regarding potential for muscle regeneration and maturation in muscular dystrophy. Cell Struct. Funct. 33, 163–169 10.1247/csf.08022 18827405

[B39] Zhao Y. Ransom J. F. Li A. Vedantham V. von Drehle M. Muth A. N. (2007). Dysregulation of cardiogenesis, cardiac conduction, and cell cycle in mice lacking miRNA-1-2. Cell 129, 303–317 10.1016/j.cell.2007.03.030 17397913

